# Study of a Crosstalk Suppression Scheme Based on Double-Stage Semiconductor Optical Amplifiers

**DOI:** 10.3390/s24196403

**Published:** 2024-10-02

**Authors:** Xintong Lu, Xinyu Ma, Baojian Wu

**Affiliations:** Key Laboratory of Optical Fiber Sensing and Communication Networks, Ministry of Education, School of Information and Communication Engineering, University of Electronic Science and Technology of China, Chengdu 611731, China; 202222011216@std.uestc.edu.cn (X.L.); 202311012307@std.uestc.edu.cn (X.M.)

**Keywords:** semiconductor optical amplifier, crosstalk suppression, optical phase conjugation, mode division multiplexing

## Abstract

An all-optical crosstalk suppression scheme is desirable for wavelength and space division multiplexing optical networks by improving the performance of the corresponding nodes. We put forward a scheme comprising double-stage semiconductor optical amplifiers (SOAs) for wavelength-preserving crosstalk suppression. The wavelength position of the degenerate pump in the optical phase conjugation (OPC) is optimized for signal-to-crosstalk ratio (SXR) improvement. The crosstalk suppression performance of the double-stage SOA scheme for 20 Gb/s quadrature phase shift keying (QPSK) signals is investigated by means of simulations, including the input SXR range and the crosstalk wavelength deviation. For the case with identical-frequency crosstalk, the double-stage SOA scheme can achieve equivalent SXR improvement of 1.5 dB for an input SXR of 10 dB. Thus, the double-stage SOA scheme proposed here is more suitable for few-mode fiber systems and networks.

## 1. Introduction

Wavelength division multiplexing (WDM) technology is widely used in optical transport networks for large-capacity transmission. To further improve the spectral efficiency of fibers, space division multiplexing (SDM) technologies based on few-mode and/or multi-core fibers are desirable for future optical networks. However, the crosstalk between spatial channels is also introduced by mode coupling [1,2]. In any case, it is essential to achieve a sufficiently low crosstalk for error-free transmission in communication networks. The multi-input multi-output (MIMO) equalization technique for crosstalk compensation has been developed in the electronic domain [3,4,5], but has not been applied to optical switching nodes due to its opaque process, algorithmic complexity, and low response.

To mitigate channel crosstalk in optical nodes, all-optical crosstalk suppression schemes are more attractive [6]. All-optical signal processing is usually based on nonlinear effects such as cross-phase modulation (XPM) and four-wave mixing (FWM) in highly nonlinear fibers (HNLFs), semiconductor optical amplifiers (SOAs), periodically poled lithium niobate (PPLN), and so on [7,8,9,10,11]. For example, the data-pump FWM and the mid-span pump-phase shifting (MPPS) techniques in HNLFs were used for crosstalk mitigation [12,13,14]. However, these HNLF-based schemes require a high input power and are limited by the simulated Brillouin scattering effect, with very low power efficiency.

By contrast, the nonlinear SOA-based crosstalk suppression schemes relax the requirements regarding the input power and are more suitable for optical networks [15]. Ku et al. demonstrated homodyne crosstalk suppression using an SOA-based Mach–Zehnder interferometric (MZI) wavelength converter for non-return-to-zero (NRZ) signals [16]. Both arms of the MZI structure must retain high symmetry. In addition, the wavelength conversion results in the use of spectral resources. In [17], the increased saturation in SOAs was used to limit the amplitude fluctuation of amplitude-modulated signals caused by the crosstalk. The optical phase conjugation (OPC) conversion in SOAs is also useful for the amplitude or phase regeneration against amplified spontaneous emission (ASE) noise and fiber nonlinearity [18].

In this paper, we utilize the OPC process in SOAs to alleviate the degradation of quadrature phase shift keying (QPSK) signals introduced by the channel crosstalk and propose a double-stage SOA scheme for wavelength-preserving crosstalk suppression. In the degenerate FWM (DFWM) process, four wavelength combinations are simulated by the VPI Design Suite, and the pump wavelength is optimized for signal-to-crosstalk ratio (SXR) improvement. The crosstalk suppression performance of the double-stage SOA scheme, dependent on the input SXR range and crosstalk wavelength deviation, is investigated for 20 Gb/s QPSK signals. Furthermore, this double-stage SOA scheme can also be used to suppress the identical-frequency crosstalk, with potential applications in mode division multiplexing (MDM) transmission networks.

The remainder of this paper is organized as follows. Section 2 describes the DFWM process of the signal and crosstalk in nonlinear SOAs, and the SXR improvement for four wavelength combinations is simulated by the VPI Design Suite. In Section 3, we put forward the double-stage SOA crosstalk suppression scheme and investigate the error vector magnitude (EVM) reduction provided by two configurations for 20 Gb/s QPSK signals, dependent on the pump-to-signal power ratio (PSPR), the input SXR range, and the crosstalk wavelength deviation. The application of the double-stage SOA scheme for identical-frequency crosstalk suppression in MDM nodes is discussed in Section 4. Finally, the conclusions are drawn in Section 5.

## 2. Optimization of Pump Wavelength in Nonlinear SOAs for Crosstalk Suppression

### 2.1. DFWM Process in Nonlinear SOAs

Nonlinear SOAs can be widely used in all-optical signal processing, such as self-phase modulation (SPM)-based chirp compensation, XPM-based wavelength conversion, and FWM-based optical regeneration. In addition, the effects of carrier density pulsations (CDP), carrier heating (CH), and spectral hole burning (SHB) occur in the SOAs [19].

**Figure 1:**
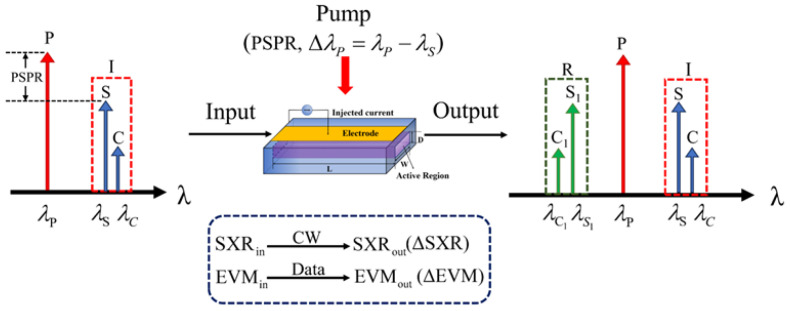
The schematic diagram of the DFWM process in SOAs.

The DFWM process in nonlinear SOAs is shown in Figure 1. The input degraded signal (I) is composed of an ideal signal (*S*) and crosstalk (*C*). The input *SXR* is defined as the ratio of the ideal signal power ($P_{S}$) to the crosstalk power ($P_{C}$)—that is, $SXR_{in}=P_{S}/P_{C}$. At the same time, a single co-polarized pump (*P*) is injected into the SOA for the DFWM interaction, in which the conjugation of the input degraded signal is regarded as the regenerated signal (R). The crosstalk suppression performance can be simulated by means of continuous waves (CWs) and pulse data and characterized by the *SXR* improvement (∆*SXR*) and EVM change (∆EVM), respectively.

Here, we firstly consider the OPC process of CWs, and the case with QPSK data will be simulated in the next section. The simulation model of the nonlinear SOA is based on the module of “SOA_TLM” given in the VPI Design Suite 9.8 software, and the detailed DFWM process is simulated by means of the transmission-line modeling (TLM) approach. The typical simulation parameters are described in Table 1.

### 2.2. SXR Improvement of Four Wavelength Combinations for a Single SOA

For the case with CWs, the conversion gains of the signal and crosstalk after OPC conversion can be obtained as follows [21]:

(1) $$\eta_{S}=\frac{{\left|A_{S_{1}}(z=L)\right|}^{2}}{{\left|A_{S}(z=0)\right|}^{2}}$$

(2) $$\eta_{C}=\frac{{\left|A_{C_{1}}(z=L)\right|}^{2}}{{\left|A_{C}(z=0)\right|}^{2}}$$

where $A_{i}(z,t)$ (*i* = *S*, *C*, *S*_1_, *C*_1_) are the complex envelopes of the optical beams.

Thus, the output signal-to-crosstalk ratio ($SXR_{out}$) is dependent on the input signal-to-crosstalk ratio ($SXR_{in}$), namely

(3) $$SXR_{out}=\frac{\eta_{S}}{\eta_{C}}SXR_{in}$$

Then, the crosstalk suppression performance in terms of the *SXR* improvement after OPC conversion (∆*SXR*) can be expressed by $\eta_{S}$ and $\eta_{C}$ that is,

(4) $$\Delta SXR=\frac{SXR_{out}}{SXR_{in}}=\frac{\eta_{S}}{\eta_{C}}$$

The Kerr nonlinearity and free-carrier dispersion (FCD) effect in the DFWM interaction induce the perturbation of the refractive index, and the conversion gains of the signal and crosstalk in the OPC process are closely related to their respective powers and wavelength positions [22]. Next, we will discuss the dependence of ∆SXR on the wavelength positions of the pump and crosstalk relative to the signal to optimize the SOA-based crosstalk suppression.

**Figure 2:**
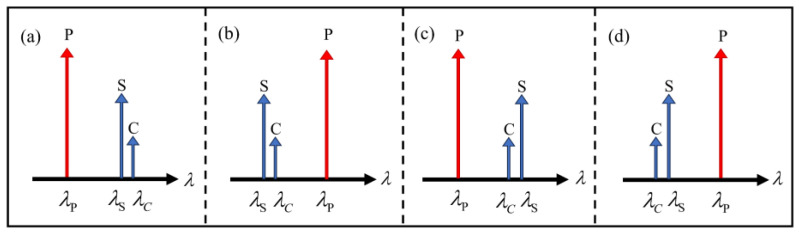
Four input combinations of the pump, signal and crosstalk wavelengths. (**a**) $\lambda_{PSC}$; (**b**) $\lambda_{SCP}$; (**c**) $\lambda_{PCS}$; (**d**) $\lambda_{CSP}$.

The ideal signal is set at 1550.52 nm with an input power of −2 dBm, and the crosstalk power is −12 dBm—that is, $SXR_{in}$ = 10 dB. The pump and crosstalk wavelengths deviate from the ideal signal, and the other parameters are taken as the default values as listed in Table 1, unless otherwise specified. Taking the ideal signal wavelength as a reference, we define the pump wavelength deviation ($\Delta \lambda_{P}=\lambda_{P}-\lambda_{S}$) and the crosstalk wavelength deviation ($\Delta \lambda_{C}=\lambda_{C}-\lambda_{S}$) to investigate the wavelength dependence of the crosstalk suppression performance in a single SOA, in which $\left|\Delta \lambda_{C}\right|$ is far less than $\left|\Delta \lambda_{P}\right|$. Thus, according to the wavelength deviations, there exist four input combinations, namely (1) $\lambda_{PSC}$, (2) $\lambda_{SCP}$, (3) $\lambda_{PCS}$, and (4) $\lambda_{CSP}$, as shown in Figure 2. For example, $\lambda_{PSC}$ denotes the case with $\lambda_{P}<\lambda_{S}<\lambda_{C}$, and so on.

**Figure 3:**
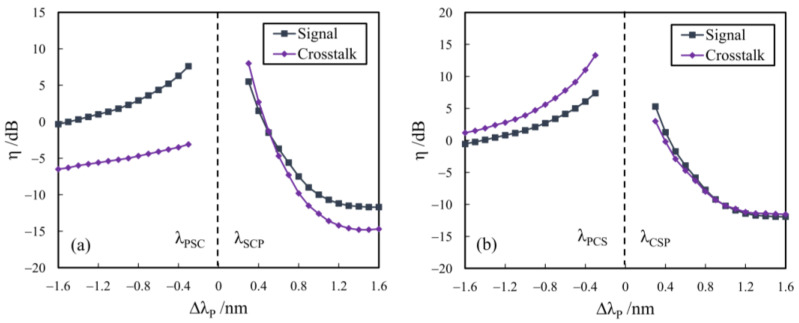
The dependences of $\eta_{S}$ and $\eta_{C}$ on $\Delta \lambda_{P}$ for (**a**) $\Delta \lambda_{C}$ = 0.08 nm and (**b**) $\Delta \lambda_{C}$ = −0.08 nm.

**Figure 4:**
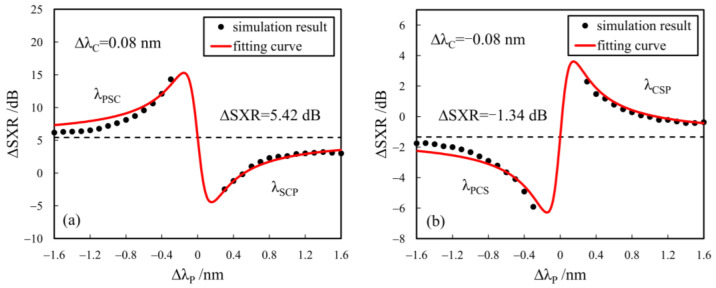
The dependences of ∆*SXR* on $\Delta \lambda_{P}$ along with their fitting curves for (**a**) $\Delta \lambda_{C}$ = 0.08 nm and (**b**) $\Delta \lambda_{C}$ = −0.08 nm.

Figure 3a and Figure 3b show the dependences of $\eta_{S}$ and $\eta_{C}$ on $\Delta \lambda_{P}$ for the cases with $\Delta \lambda_{C}$ = ±0.08 nm, respectively, where the input *SXR* ($SXR_{in}$) is 10 dB and the input powers of the signal and pump are −2 dBm and 6 dBm, respectively, with a pump-to-signal power ratio (PSPR) of 8 dB. From Figure 3, the conversion gains for both the signal ($\eta_{S}$) and crosstalk ($\eta_{C}$) reduce with the increase in |$\Delta \lambda_{P}$| due to larger wavelength detuning. The conversion gains of the signal and crosstalk are also dependent on the change in the refractive index induced by the free-carrier dispersion and Kerr nonlinearity, resulting in their discrepancy in SXR improvement (∆*SXR*). The variations of ∆*SXR* with $\Delta \lambda_{P}$ are plotted in Figure 4a,b, and the corresponding fitting curves can be expressed as follows:

(5) $$\left\{\begin{array}{cc} \Delta SXR=\frac{5.961\times 10^{-6}\times \Delta \lambda_{P}}{-1.946\times 10^{-6}\times \Delta {\lambda_{P}}^{2}-4.678\times 10^{-8}}+5.42, & \Delta \lambda_{C}=0.08\;nm\\ \Delta SXR=\frac{9.745\times 10^{-5}\times \Delta \lambda_{P}}{6.658\times 10^{-5}\times \Delta {\lambda_{P}}^{2}+1.454\times 10^{-6}}-1.34, & \Delta \lambda_{C}=-0.08\;nm \end{array}\right.$$

From Figure 4a, for the cases of $\lambda_{PSC}$ and $\lambda_{SCP}$ with $\Delta \lambda_{C}$ > 0, the ∆*SXR*s increase with $\Delta \lambda_{P}$ and their ∆*SXR* curves are, respectively, larger and lower than the limiting value of 5.42 dB, corresponding to a sufficiently large $\left|\Delta \lambda_{P}\right|$ according to Equation (5). In contrast, for the cases of both $\lambda_{PCS}$ and $\lambda_{CSP}$ with $\Delta \lambda_{C}$ < 0, as shown in Figure 4b, the ∆*SXR*s decrease with the increase in $\Delta \lambda_{P}$ and their ∆*SXR* curves are, respectively, lower and larger than the limiting value of −1.34 dB.

Clearly, among the four combinations, the case with $\lambda_{PSC}$ has the best performance in SXR improvement, and the next is the case with $\lambda_{CSP}$. They have the common laws that (1) the signal is closer to the pump than the crosstalk in wavelength and (2) the *SXR* improvement decreases with the increase in $\left|\Delta \lambda_{P}\right|$, which are both related to the phase mismatching in the DFWM process.

## 3. The Double-Stage SOA Scheme for Crosstalk Suppression

According to the OPC process in the single SOA, we put forward a wavelength-preserving crosstalk suppression scheme based on the double-stage SOAs, including two specific configurations. The simulation parameters used here are the same as in Table 1.

### 3.1. The Double-Stage SOA Scheme

**Figure 5:**
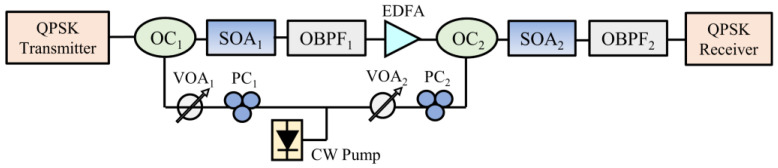
The double-stage SOA-based crosstalk suppression scheme for QPSK signals.

The double-stage SOA-based crosstalk suppression scheme is shown in Figure 5. QPSK signals are generated by an in-phase/quadrature (I/Q) modulator in the optical transmitter and coherently demodulated at the optical receiver after regeneration. Each SOA stage is composed of an optical coupler (OC), a single SOA, and an optical bandpass filter (OBPF). An erbium-doped fiber amplifier (EDFA) is located between the two SOA stages to increase the converted power. The two SOAs share the same CW pump source. The detailed simulation process is described as follows: the degraded signal is generated from the QPSK transmitter, in which the ideal signal is fixed at 1550.52 nm and an input power of −2 dBm, and the SXR and crosstalk deviation ($\Delta \lambda_{C}$) are adjusted. The degraded signal and the pump are injected to SOA_1_ through OC_1_, and the generated conjugated light can be obtained from OBPF_1_. For the purpose of wavelength preservation, SOA_2_ has to be used for a second phase conjugation at an input signal power of −2 dBm, just like SOA_1_. The pump-to-signal power ratios (PSPR_1_ and PSPR_2_) of SOA_1_ and SOA_2_ can be adjusted by the variable optical attenuators (VOA_1_ and VOA_2_) for improving the performance of crosstalk suppression. The regenerated signal at 1550.52 nm is filtered out by the following OBPF_2_. The OBPFs are of the first-order Bessel function with a 3 dB bandwidth double the data rate. The quality of the regenerated QPSK signals is coherently detected by the QPSK receiver.

According to the analysis carried out in Section 2.2, we focus on two configurations—that is, SOA_1_ and SOA_2_ are set at (1) $\lambda_{PSC}$ + $\lambda_{CSP}$ or (2) $\lambda_{CSP}$ + $\lambda_{PSC}$, respectively.

### 3.2. Optimal PSPR of SOA_1_ for QPSK Signals

**Figure 6:**
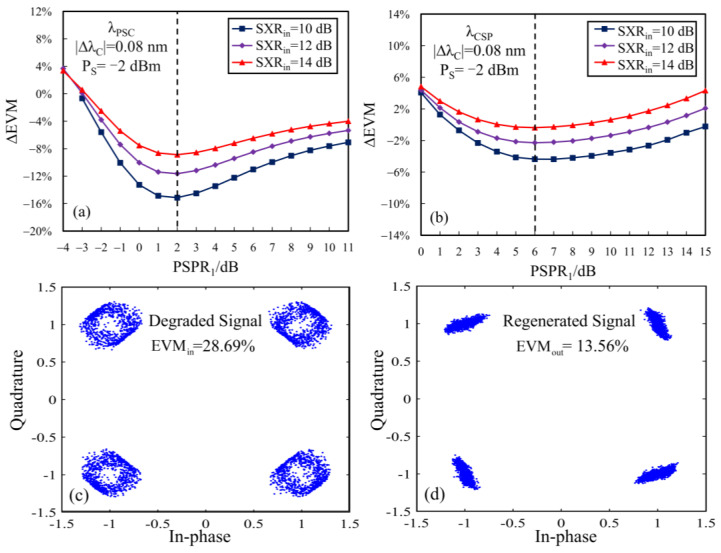
The EVM change dependent on PSPR_1_ for (**a**) $\lambda_{PSC}$ and (**b**) $\lambda_{CSP}$, and the constellation for (**c**) the degraded signal and (**d**) the regenerated signal.

Here, we simulate the crosstalk suppression performance of SOA_1_ for the 20 Gb/s QPSK signal provided that $\left|\Delta \lambda_{C}\right|$ = 0.08 nm and $\left|\Delta \lambda_{P}\right|$ = 0.8 nm. The 3 dB bandwidth of OBPF_1_ after SOA_1_ is set at 0.32 nm. Obviously, in this case, the crosstalk cannot be eliminated by OBPF_1_. Figure 6a and Figure 6b illustrate the EVM changes (∆EVM = EVM_out_ − EVM_in_) dependent on the pump-to-signal power ratio when SOA_1_ is set at $\lambda_{PSC}$ and $\lambda_{CSP}$, respectively. In Figure 6a and Figure 6b, we can see that the maximum EVM reduction occurs at the optimal PSPR_1_ of 2 dB and 6 dB, respectively, which is almost independent of $SXR_{in}$. For the case with $\lambda_{PSC}$, the constellation diagrams of the degraded and regenerated QPSK signals at the optimal PSPR of 2 dB and the input *SXR* of 10 dB are demonstrated in Figure 6c,d. Clearly, the crosstalk degradation on the constellation diagrams is effectively suppressed, especially in amplitude noise, and the EVM parameter is reduced to 13.56% from 28.69%. It can be seen that the single OPC-based SOA acts as the function of phase persevering amplitude regeneration (PPAR). In the following simulation, SOA_1_ is always set at the corresponding optimal PSPR_1_.

### 3.3. The EVM Performance of the Double-Stage SOA Scheme

The optimization of PSPR_2_ for the second-stage SOA (SOA_2_) differs from the case for SOA_1_, because the signal structure input into SOA_2_ is nonlinearly changed by SOA_1_; this leads to the dependence of the optical PSPR_2_ on the specific configuration. For comparison, PSPR_2_ is fixed at 6 dB for both configurations. Next, we investigate the crosstalk suppression performance of the double-stage SOA scheme for QPSK signals, in which $SXR_{in}$ and $\left|\Delta \lambda_{C}\right|$ are changed by adjusting the crosstalk power and wavelength, respectively.

**Figure 7:**
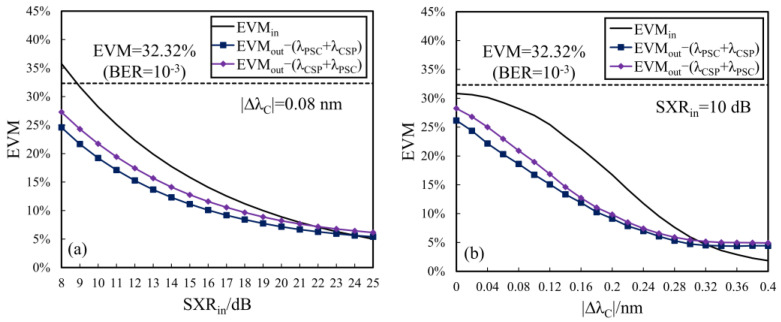
The EVM curves dependent on (**a**) $SXR_{in}$ and (**b**) $\left|\Delta \lambda_{C}\right|$.

Figure 7a shows the variations in the input and output EVMs (EVM_in_ and EVM_out_) with $SXR_{in}$ for two configurations when $\left|\Delta \lambda_{C}\right|$ = 0.08 nm. For the 20 Gb/s QPSK system, the EVM threshold is 32.32%, corresponding to the bit error rate (BER) of 10^−3^ in the absence of forward error correction (FEC). It can be seen from Figure 7a that (1) with the increase in $SXR_{in}$, the crosstalk suppression performance denoted by $\left|\Delta \mathrm{EVM}\right|$ becomes gradually smaller; (2) the configuration of ($\lambda_{PSC}$ + $\lambda_{CSP}$) is superior to the other ($\lambda_{CSP}$ + $\lambda_{PSC}$) in EVM reduction for any given $SXR_{in}$; and (3) the crosstalk within the range of $SXR_{in}$ ≤ 24 dB can be suppressed by using the double-stage SOA scheme with ($\lambda_{PSC}$ + $\lambda_{CSP}$).

The dependences of EVM_in_ and EVM_out_ on $\left|\Delta \lambda_{C}\right|$ at $SXR_{in}$ = 10 dB are plotted in Figure 7b. From Figure 7b, we can see that both EVM_in_ and the output EVMs reduce with the increase in $\left|\Delta \lambda_{C}\right|$, and the EVM reduction reaches its maximum value when $\left|\Delta \lambda_{C}\right|$ = 0.12 nm, corresponding to ∆EVM = −10.35% and −8.58% for the configurations of ($\lambda_{PSC}$ + $\lambda_{CSP}$) and ($\lambda_{CSP}$ + $\lambda_{PSC}$), respectively. For the homodyne crosstalk ($\left|\Delta \lambda_{C}\right|$ = 0 nm), both configurations can also perform the function of EVM reduction. Certainly, the crosstalk suppression function will become invalid for $\left|\Delta \lambda_{C}\right|$ ≥ 0.32 nm due to the XPM-induced degradation in the OPC process.

The two double-stage SOA configurations have some differences in the OPC orders and the PSPR parameters of the first-stage SOA. The PSPR_1_ of the first configuration ($\lambda_{PSC}$ + $\lambda_{CSP}$) is lower than the other case ($\lambda_{CSP}$ + $\lambda_{PSC}$). In a word, the double-stage SOA scheme with ($\lambda_{PSC}$ + $\lambda_{CSP}$) has better crosstalk suppression performance than that with ($\lambda_{CSP}$ + $\lambda_{PSC}$) for any input degraded signal, and the former is more desirable for the large-scale optical MDM network from the low power consumption point of view.

## 4. Application of the Double-Stage SOA Scheme to the MDM System with Identical Frequency Crosstalk

**Figure 8:**
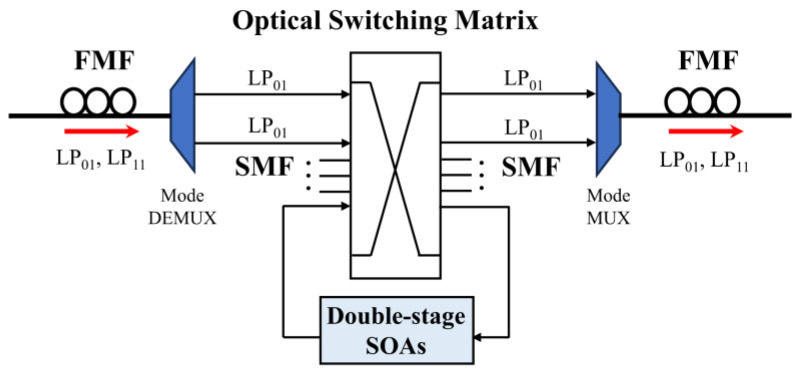
Feedback crosstalk suppression scheme for optical switching nodes based on MDM.

It is well known that the combination of various multiplexing technologies, such as WDM, MDM, and polarization division multiplexing (PDM), can greatly enhance the transmission capacity of future optical transport networks [23]. The resulting multidimensional optical switching nodes (MD-OSN) should support the mode switching function at the same wavelength. One kind of MD-OSN architecture is based on the single-mode domain—that is, all high-order modes are demultiplexed to the fundamental modes for mode switching and then re-multiplexed to other high-order modes after optical switching [24]. Because of the imperfect mode demultiplexing process, the modal crosstalk may take place at the same frequency as the signals ($\left|\Delta \lambda_{C}\right|$ = 0 nm). Taking into account the MDM system with two linearly polarized modes (LP_01_ and LP_11_), the high-order mode LP_11_ is converted into the fundamental mode by a mode demultiplexer (DEMUX) for optical switching. In course, the crosstalk from the LP_01_ mode leads to the LP_11_-mode signal degradation. To reduce the modal crosstalk, we can employ a feedback crosstalk suppression scheme—that is, the degraded signal is switched to the double-stage SOA structure, and then the regenerated signal is re-directed to its destination through the single-mode switch matrix, as shown in Figure 8.

**Figure 9:**
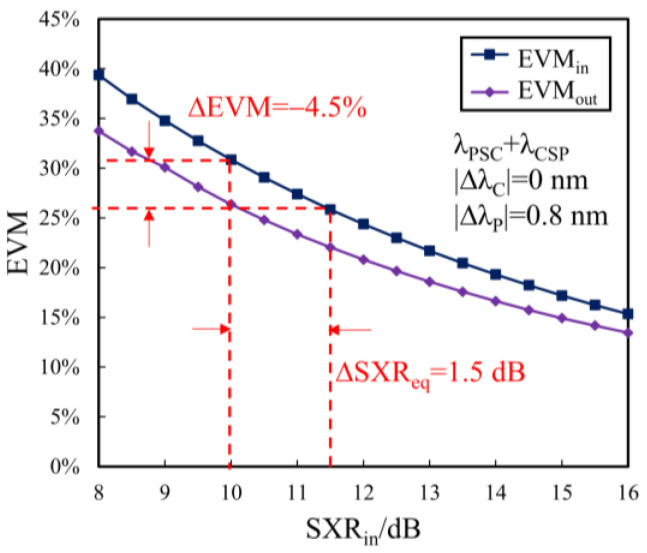
Double-stage SOAs for crosstalk suppression based on MDM.

In the application scenario with identical-frequency crosstalk, we here adopt ($\lambda_{PSC}$ + $\lambda_{CSP}$) to evaluate the crosstalk suppression performance provided by the equivalent SXR improvement ($\Delta SXR_{eq}$), which can be determined by the EVM reduction, as shown in Figure 9. Figure 9 also gives the EVMs dependent on the input SXR before and after the double-stage SOAs for the case with $\left|\Delta \lambda_{C}\right|$ = 0 nm; the other parameters are the same as in Figure 7a. From Figure 9, when $SXR_{in}$ = 10 dB, the output EVM reduces to 26.36% from the input EVM of 30.86% for 20 Gb/s QPSK signals, corresponding to ∆EVM = −4.5% and $\Delta SXR_{eq}$ = 1.5 dB.

**Figure 10:**
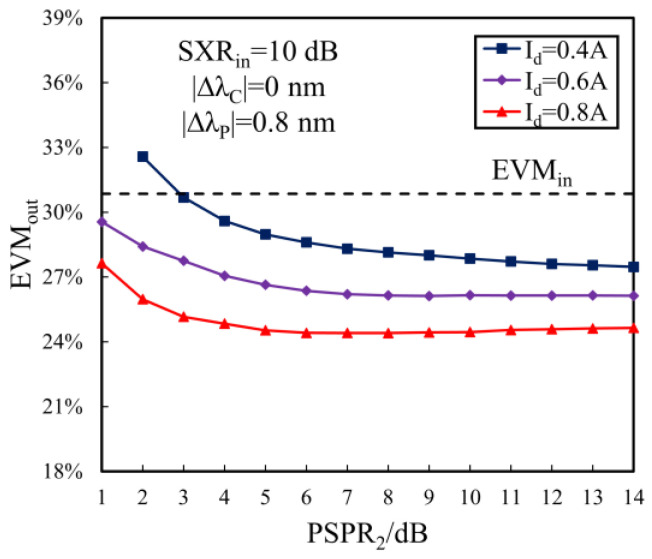
The output EVM dependent on PSPR_2_ for different driving currents of SOA_2_.

Furthermore, the modal crosstalk suppression performance can be further improved by optimizing the driving current and PSPR_2_ of SOA_2_. Figure 10 shows the output EVM dependent on PSPR_2_ at the different currents. From Figure 10, the output EVM reduces with the increase in PSPR_2_ and the driving current I_d_, and the EVM_out_ reduces to 24.41% when PSPR_2_ = 6 dB and I_d_ = 0.8A, corresponding to $\Delta SXR_{eq}$ = 2.2 dB. Clearly, the double-stage SOA scheme is desirable for the MDM transmission from the perspective of crosstalk suppression.

Then, we perform a comparison between all-optical crosstalk suppression schemes. Table 2 lists several crosstalk suppression schemes based on HNLFs and SOAs in terms of modulation format, crosstalk deviation, and wavelength preservation. From Table 2, we can see that Refs. [13,14] presented HNLF-based regeneration techniques with crosstalk deviation of more than 0.4 nm, which have disadvantages regarding wavelength conversion. The SOA-MZI technique given in Ref. [16] can perform homodyne crosstalk mitigation but needs a high-precision device due to the interference structure. A single SOA can also be used to suppress the crosstalk based on its gain saturation regardless of the waveform distortion at high powers [17]. By contrast, the double-stage SOA scheme in this paper possesses wavelength preservation and a good phase-persevering amplitude regeneration function, along with a wider regenerative range of $\left|\Delta \lambda_{C}\right|$ ≤ 0.32 nm for the 20 Gb/s QPSK signals. Therefore, our proposal is desirable for crosstalk suppression in multidimensional optical switching nodes. Recently, emerging nanomaterials such as carbon nanotubes have attracted more and more attention due to their excellent optical nonlinearity, ultrafast response, and high integration [25,26,27]. In principle, the nonlinearity and tunability of their refractive index can also be expected to achieve all-optical crosstalk suppression, regardless of large losses.

## 5. Conclusions

In this paper, four DFWM combinations in a single SOA are investigated to optimize the pump wavelength. We propose the double-stage SOA scheme for wavelength-preserving crosstalk suppression. The crosstalk suppression performance for 20 Gb/s QPSK signals is simulated. It is shown that the double-stage SOA configuration of ($\lambda_{PSC}$ + $\lambda_{CSP}$) displays the best performance in crosstalk suppression, with a regenerative input SXR range of $SXR_{in}$ ≤ 24 dB and a crosstalk deviation of $\left|\Delta \lambda_{C}\right|$ ≤ 0.32 nm. This scheme can achieve the equivalent SXR improvement of 1.5 dB for the identical-frequency crosstalk when the input SXR is 10 dB, and the modal crosstalk suppression performance may be further improved by appropriately increasing the driving current of SOA_2_. In summary, the double-stage SOA scheme is applicable for the MD-OSNs indispensable for future optical networks.

## Figures and Tables

**Table 1:** The default parameters in “SOA_TLM” [20].

Symbol	Description	Value	Unit
L_k_	Length of the device section	500	μm
w_k_	Width of the active region	3	μm
d_k_	Thickness of the active region	0.08	μm
I_k_	Current injected to the SOA	600	mA
n_2,k_	Nonlinear Kerr coefficient	6.2 × 10^−19^	m^2^/W
α_a_	Internal loss coefficient in active region	4 × 10^3^	m^−1^
Г_k_	Optical confinement factor for bulk section	0.3	/
Г_NL,k_	Nonlinear optical confinement factor	1	/
A_eff,k_	Effective mode areas	10^−12^	m^2^
a_lin,k_	Linear gain coefficient	2.78 × 10^−20^	m^2^
ε	Gain suppression factor	1 × 10^−23^	m^3^
α_lw_	Linewidth enhancement factor	3	/
A_k_	Linear carrier recombination coefficient	1.43 × 10^−8^	s^−1^
B_k_	Bimolecular carrier recombination coefficient	10^−16^	m^3^/s
C_k_	Auger carrier recombination coefficient	3 × 10^−41^	m^6^/s
N_0,k_	Carrier density transparency	1.5 × 10^24^	m^−3^
N_ch,k_	Reference carrier density	2 × 10^24^	m^−3^

**Table 2:** Comparison of the crosstalk suppression schemes.

Schemes	Modulation Format	Crosstalk Deviation	Wavelength Preservation	Properties
Mid-span pump phase shift in HNLF [13]	QPSK	0.4 nm	No	Using a programmable filter
Data-pump FWM-HNLF [14]	RZ	0.4 nm	No	High input powers
MZI-SOA [16]	NRZ	homodyne	No	High-precision device
Gain-saturated SOA [17]	OOK	No available	Yes	Waveform distortion at high power
Our scheme (double-stage SOAs)	QPSK	0~0.32 nm	Yes	Phase persevering amplitude regeneration function

## Data Availability

The data underlying the results presented in this paper are not publicly available at this time but may be obtained from the authors upon reasonable request.
